# COVID‐19: A catalyst for change in virtual health care utilization for persons with limb loss

**DOI:** 10.1002/pmrj.12605

**Published:** 2021-05-28

**Authors:** Melissa A. Hewitt, Douglas G. Smith, Jeffrey T. Heckman, Paul F. Pasquina

**Affiliations:** ^1^ The Center for Rehabilitation Sciences Research Uniformed Services University of Health Sciences Bethesda Maryland USA; ^2^ Walter Reed National Military Medical Center Bethesda Maryland USA; ^3^ The Henry M. Jackson Foundation for the Advancement of Military Medicine Bethesda Maryland USA; ^4^ James A. Haley VA Medical Center Tampa Florida USA; ^5^ Physical Medicine and Rehabilitation University of South Florida Tampa Florida USA

## Abstract

The dramatic uptake of virtual care, or telehealth, utilization because of COVID‐19 restrictions for persons with limb loss has led to a much greater understanding of this health care delivery method for this complex patient population. However, much is still unknown. Therefore, the authors provide a comprehensive literature review of existing evidence for virtual care delivery across the phases of amputation rehabilitation, as well as anecdotal evidence, to provide a platform for further discussion and development of research and innovative opportunities. Evidence reveals that virtual care serves as a complement to in‐person health care for individuals with limb loss because it allows for increased accessibility to these services. The authors conclude that continued use of telehealth beyond the COVID‐19 restrictions to optimize outcomes across the continuum of care for persons with limb loss is warranted.

## INTRODUCTION

The coronavirus disease of 2019 (COVID‐19) has swept the globe at unprecedented proportions and left many changes in its wake. One of those changes has been the need for rapid uptake and utilization of virtual care as the primary source of health care delivery. Before the global pandemic, virtual care or telehealth had already been gaining significant traction in the U.S. health care system, with telehealth insurance claims rising by 53% from 2016 to 2017.^1^ In response to the ongoing challenges in delivering health care during the pandemic, many of the rules that governed provider–patient interaction, privacy, documentation, and reimbursement that had previously constrained widespread use of telehealth have now been lifted.^1^, ^2^ Although many of these measures are likely temporary, it has become clear that telehealth has the potential to greatly improve health care accessibility and efficiency, especially in medical and rehabilitation specialties for persons with mobility impairments such as limb loss.

There are almost 2 million individuals living with limb loss in the United States, and an estimated 185,000 new amputations occur each year. The majority of new amputations occur in elderly persons with multiple medical comorbidities, including diabetes mellitus and peripheral vascular disease,^3^, ^4^ which only further complicates challenges with mobility and access to health care.^5^ Because of the specialized nature of caring for persons with limb loss and the mobility challenges they face, virtual care offers a customizable solution across the spectrum of amputation rehabilitation care, both before and after an amputation.^6^ Evidence of high satisfaction rates have been shown in telehealth delivery, with one study reporting 97% of individuals who received amputation related virtual care in Canada had “good” or “excellent” satisfaction ratings.^7^ In another survey, 87% of a sample of patients who underwent a telehealth wound examination thought that telehealth would be a helpful tool throughout their continued care needs.^8^ In addition to a high level of patient satisfaction, telehealth may also offer a more time‐ and cost‐effective way of delivering care.^6^, ^9^

Although the literature is clear that telehealth is not a substitute to in‐person care, many studies have shown that it can be used as a complementary tool to improve health outcomes and satisfaction for both providers and patients.^6^, ^10^ Because of the complexity of amputation rehabilitation care, it is very important that rigorous reporting clarify which aspects of the amputation care process would most benefit from the use of telehealth. Therefore, the purpose of this paper is threefold: first, to outline the phases of amputation rehabilitation for consideration of virtual care utilization; second, to review the current literature to support the virtual care environment as a complement to in‐person care; and third, to stimulate discussion for process improvement of virtual care opportunities.

## LITERATURE REVIEW

The authors discussed possible structures for examining the implementation of telehealth into the amputation care process. The authors decided on the current structure in order to extrapolate what research was currently available and where paucities were present. Upon this determination, a literature search was performed using PubMed between August and December 2020 to encompass all available papers on this topic. The primary search strategy involved utilization of the following search terms, as they encompassed the breadth of this topic: “telehealth,” “telemedicine,” “virtual care,” “amputee,” “amputation,” “limb loss,” “rehabilitation,” “support,” and “timeline.” Certain sections warranted more specific search terms such as “diabetic foot ulcers,” “telesurgery,” and “physical therapy.” In total, 73 articles were assessed for relevance, and only 51 were included. Of the 51 papers included in this review, 15 were published in the past 2 years, and 31 were published in the last 3 years. Inclusion criteria included mention of virtual care or telehealth, as well as a clinical application for individuals with limb loss; this encompassed synchronous care, asynchronous care, and remote patient monitoring (RPM). Exclusion criteria included lack of use of virtual care or telehealth and no relevance to the limb loss population; as a result 22 articles were excluded. Additionally, manual searches of article references were performed so as to elicit further articles for review. Articles spanned many different research facilities including those in the United States, Canada, Europe, and Australia. When unavailable on PubMed, authors utilized the journals provided by their institution's resource center, including, but not limited to, Elsevier, Ovid MEDLINE, Sage, and JAMA.

## DISCUSSION

### Preamputation phase

Diabetes mellitus (diabetes) and peripheral vascular disease (PVD) are two of the major causes of amputation, the third being trauma.^11^ Although a traumatic amputation is difficult to prepare for, whether using telehealth or not, chronic conditions like diabetes and PVD can be carefully monitored to slow the impact of these conditions and perhaps prevent amputation altogether. The overall 1‐year mortality rate after a diabetes‐related amputation is 24%,^8^ which may rise as high as 70% after 3 years.^12^ In individuals with an amputation secondary to PVD, 10% of transtibial amputations did not heal properly and required a subsequent higher level amputation.^8^ Thus, the main goal at this phase is to monitor and prevent deterioration of chronic conditions, especially for individuals with diabetes and PVD. One of the many ways telehealth can be integrated into this process is via RPM. This allows clinicians and support staff to monitor specific health markers, such as patient vitals, to assess progress while avoiding the need for patients to travel unnecessarily. In this way, clinicians can have greater amounts of data to analyze when patients return for in‐person treatments.

#### Diabetic foot ulcer monitoring

Diabetic foot ulcers (DFU) are very common and serious complications of diabetes. Many DFUs go untreated for an extended period of time, with up to one‐third of all DFUs never healing and resulting in amputation.^12^ Telehealth offers an opportunity for patients and providers to more easily monitor and manage DFUs before they worsen. Ploderer et al used qualitative interviews to assess participant responses to a mobile app that helped to track progression of DFUs. Participants found the objective data helpful for their own self‐care and speculated the RPM features would be useful during discussions with clinicians.^13^ However, in another study from 2019, providers expressed concerns about the integrity and true objectivity of the data obtained by patients via telehealth.^12^ To this end, Rintala et al compared a provider's wound ratings after one examination via telehealth and another examination in person.^8^ The photographs of the wounds were taken in a telehealth lab and examined by the provider. Then the provider examined that same wound in person. Overall, this led to a 92% agreement between both ratings; and providers rated 80% of wound photographs as excellent, very good, or good. Typically, the pictures that were not rated as highly were those of smaller wounds that were more difficult to photograph. Overall, the authors concluded that telehealth was a feasible alternative for examining DFUs or postamputation wounds and resulted in similar ratings as in‐person examinations.^8^ A corresponding study used telehealth to monitor DFUs during the COVID‐19 pandemic using comparable methods to the previous study. These researchers also found that telehealth allowed for adequate management of DFUs and no spread of the coronavirus.^14^

Further advancements beyond photographs for prevention of progression of DFUs are available and show promise. Galileo first discussed a thermoscope in the late 1500s, but it was not until 1957 that the field of clinical medical thermography was officially established.^15^ Modern technology has allowed thermography to be used not just in the clinical setting but also in the home. In the case of DFUs, an asymmetry in temperature of 2.22°C over at least 2 days is considered elevated risk and requires further medical attention.^16^ Various studies have looked at a temperature monitoring mat that can be kept at a patient's home.^16^, ^17^, ^18^ Frykberg et al studied 129 patients who used the mat and found high adherence, with 86% of individuals measuring their plantar temperature an average of three times a week, and 88% stating the mat was easy to use. Additionally, researchers found that 97% of all DFUs were found with the thermography mat, and on average they were detected 37 weeks before they were detected in person, in the clinic. Gordon et al analyzed the same study set, this time specifically focusing on high‐risk individuals such as those with a recent wound or amputation. Even so, the researchers found that the thermography mat was effective in detecting DFUs, and for some patients those DFUs were predicted before they presented clinically.^17^ Other studies have looked into different forms of thermography. For example, an experiment done by Reyzelman et al looked at temperature monitoring socks and whether they could be integrated into routine care. The research team found that not only were the sensors in the socks reliable and accurate, but the patients found the socks and accompanying app interface easy to navigate.^18^

Although photographic and thermographic monitoring have shown promise, a primary concern in treating highly complex patients relates to the impact of less face‐to‐face interaction between patients and their caregivers. Although Wallace et al found that 73% of participants were interested in using a mobile health app to check their feet,^12^ there are some drawbacks to this approach. For example, much can be learned from the body language of the patient and interaction with their provider that is difficult to fully appreciate during a virtual encounter. This concern is reinforced by one study from 2015, which reported that patients with DFUs who were monitored via telehealth had significantly higher mortality rates than those monitored in person.^19^ Yet, there was no difference between the in‐person and telehealth groups in terms of the number of patients healed or the number of patients who later needed a limb amputated.^19^ The authors could not find an explanation for this conclusion given the variables they measured. As such, future research should explore what may cause higher mortality rates in individuals being monitored via telehealth and how to mitigate these outcomes.

It is also important to note that the rapid rate at which technology advances may play a factor in future outcomes. For example, Rasmussen et al. used telephone calls or online written consultations supplemented by photographs.^19^ However, video conferencing is increasingly becoming popular and may add a more personalized aspect to virtual care. Therefore, it is important to remember that although these studies add to the current body of knowledge, future research will need to extrapolate as to how newer communication platforms and technologies affect outcomes.

There appear to be clear and distinct advantages for monitoring and management of DFUs using virtual care as a complement to in‐person care. It will require a measured approach to create the confluence of these delivery options that results in the highest quality of care to prevent amputation.

#### PVD monitoring

Treatment strategies for prevention of amputation due to PVD typically include at least two prongs: consistent exercise and tobacco cessation. Recent studies have looked into the use of telehealth to encourage patient adherence to both of these practices. One study examined wearable activity trackers in an at‐home program designed to encourage individuals with PVD to increase their physical activity levels.^20^ The authors, McDermott et al, periodically reached out to the participants via phone call, which replaced in‐person visits after the first month, in order to check on participant progress and adherence to the program. However, at 9‐month follow‐up, both individuals with the activity tracker and individuals in the control group saw no improvement in walking performance as measured by the 6‐minute walk test.^20^ McDermott et al believed this was because of the lack of in‐person feedback after the first month and recommended future studies and interventions contain an in‐person component. Thus, it would seem that the literature supports telehealth as a complement, and not a replacement, for patient monitoring of PVD. Another study utilized an online application that would allow patients to monitor various aspects of their health, including heart rate and blood pressure, in one central location that providers also had access to.^21^ This was meant to promote lifestyle changes and help patients self‐manage their health from their homes. Initial survey data seemed promising and patients thought the system would be “useful, beneficial, and rewarding to use”; however, further statistical analysis showed that the amount of effort people expended using the system did not correlate to behavior change. Similar to the previous study, Aria and Archer interpreted these results to indicate that telehealth should be used in conjunction with in‐person care and cannot be utilized as a complete substitution.^21^

Tobacco cessation is also an important aspect of disease prevention, leading investigators to explore the use of telehealth and virtual care to help promote decreased use of tobacco. One study by Moore et al tested the validity of monitoring tobacco usage using remote cotinine swabs. The authors collected two swabs from each participant, one that was immediately analyzed and one that the researchers mailed to their own laboratories a few days later and from a different location than the testing site. This was meant to imitate the possible range of durations from initial collection to final interpretation if patients were to mail in their swab kits after using them at home. Moore et al found that the final results of the swab did not change as a result of the time from collection to mailing, and thus this was a reliable method for quantifying tobacco usage in patients.^22^ Future studies should also examine whether a telehealth visit with a provider, combined with the cotinine swabs, can cause behavior change in individuals attempting to reduce their tobacco intake.

Additional studies have examined a variety of other technological approaches to tobacco cessation. For example, Comello and Porter created a smart cigarette case that would track how many cigarettes were used and provide feedback to the user on their progress toward quitting smoking. Although participants generally found this technology promising, they recommended additional modifications, such as a smaller and more customizable case.^23^ Chahar et al examined the feasibility of text messages as a reminder to individuals who wanted to quit smoking and reported that participants found the texts to be understandable and of high appeal.^24^ Finally, Pulverman and Yellowlees reviewed a variety of mobile health apps used for tobacco cessation, and although there are a plethora of available mHealth apps, there remains a lack of scientific evidence supporting their efficacy. In addition, the authors reported that there remain a number of major drawbacks to tobacco cessation applications, including their lack of in‐person communication between patients and providers and the steep learning curve required to use the technology.^25^

In summary, there appears to be great potential benefit in using telehealth technologies to promote exercise and assist with tobacco cessation for individuals at risk for amputation due to PVD, but further research is needed to best guide clinical practice and optimize its efficacy.

### Surgical decision making and inpatient care phase

Although traditionally used to augment outpatient care, telehealth is gradually being introduced to the inpatient setting, and may have a significant role in the care of individuals undergoing amputation surgery. The decision to amputate is a very difficult one, requiring careful thought from both patients and providers. Remote consultations to experts may help in this process. Historically, surgeons have used telephone communication to discuss difficult cases with outside experts and colleagues. Newer platforms with video capability augment this practice by allowing practitioners to transmit clinical photographs and videos that can assist in decisions such as surgical approach, amputation level, or whether the amputation surgery should be conducted at a specialized facility. The evolution from telephone to video chat has not only improved communication but added a dimension of “personalization” needed in today's patient‐focused care approach. Furthermore, the technology allows teams of clinical experts from different hospitals to directly communicate with patients and families in order to discuss and develop plans involving continuum of care decisions and postsurgical rehabilitation, such as coordinating home therapy and equipment needs (personal experience of the authors). Further research is needed to understand the impact of these technologies and identify best practices for implementation across different health care systems.

With regard to the intersection between telehealth and surgery, even before the COVID‐19 pandemic, there have been a number of pilot trials in “telesurgery,” primarily involving spinal surgery and prostatectomies.^2^ To date, there have been no reported cases of “telesurgery” being applied to amputation, though newer technologies may allow further investigation. For example, such studies might include a livestream video of an expert surgeon observing amputation surgery and providing “real‐time” input on approaches to soft tissue or peripheral nerve management, including targeted muscle reinnervation.^2^, ^26^ Anecdotal evidence from one of the authors (D.G.S.) suggests that this may be a common practice among surgeons and trainees, as he himself has been remotely called into the operating room.

There are also a number of articles that have examined the use of smartphones and direct messaging in a variety of inpatient settings. For example, Dala‐Ali et al provided an overview of the uses of smartphones for surgeons, noting that in addition to text messages, smartphones can send images and videos as well as provide educational tools and apps.^27^ Another study by Koparal et al examined a group of maxillofacial surgeons using WhatsApp to communicate among the team and provide consultations between surgeons. Out of all consultations, 62.8% were conducted via messages, with the majority engaging a consultant who was not in the hospital.^28^ Similarly, Paik et al found that plastic surgery telehealth consult response time was, on average, 4.5 minutes, whereas in‐person response time was an average of 56 minutes. The immense difference between these two response times shows how rapidly telehealth consultations can take place and therefore greatly decreasing wait times for patients. Additionally, there was a 90.5% agreement rate between remote and in‐person consults with a board‐certified plastic surgeon.^29^ These studies provide further evidence that telehealth may play an important role in expanding surgical capabilities in the future.

Telehealth may also be helpful in augmenting inpatient pain management. A study by Spiegel et al examined the use of virtual reality to supplement pain management for hospitalized patients. Researchers found that although patients reported higher levels of satisfaction, there was only a small statistical reduction in pain and no difference in opioid consumption between the intervention and control groups.^30^ Therefore, further research is needed to explore the role of telehealth and virtual applications in improving inpatient pain management.

In summary, there appears to be a variety of potential uses for telehealth to improve inpatient care, including individuals with amputation, which should be further explored.

### Postoperative care and preprosthetic training phase

After surgery the postoperative and preprosthetic training phase begins. This phase focuses on incision site healing, edema control, pain management, and the prevention of contractures or injury to the residual limb. In addition, rehabilitation professionals will engage with patients to improve mobility with either crutches or a wheelchai, as well as independence with activities of daily living. Home or vehicle modifications may also need to be made to enhance successful reintegration back into the home and community. Many individuals are discharged directly home for residual limb care and healing, while others are often discharged directly to inpatient rehabilitation. Although telehealth can be useful in both settings, it should be emphasized for individuals who are discharged directly home and may have many unmet needs such as contracture prevention and difficulties with transfers.

#### Wound care management

Surgical site infections represent the most common cause of hospital readmission after surgery.^31^ This coupled with the fact that hospital length‐of‐stay continues to decrease, add additional emphasis on the need to develop better ways to monitor wound healing.^31^ New virtual capabilities have the potential to bridge this gap, with several studies already examining this potential. Gunter et al developed a mHealth application, and examined its feasibility in monitoring postoperative wounds.^31^ Researchers found that both patients and providers were able to adhere to the monitoring requirements fairly easily. Patients submitted pictures of their wounds as well as any information about their symptoms via an internally developed app. Overall patient submission rate was 90.2%, with 91.9% of all submissions reviewed within 24 hours, and the average length of time between submission and examination was 9.7 hours. Providers were enthusiastic about the mHealth monitoring system but concerned about the additional time commitment to their already busy schedules. To address these concerns, the authors recommended assigning a dedicated care team specifically to monitor these wounds so as not to overburden providers.^31^ Another study by Rintala et al compared in‐person versus telehealth assessment of incisions following amputation surgery. The researchers found that there was a 92% agreement between in‐person and telehealth examinations, leading to the conclusion that there is a high level of reliability when comparing virtual to in‐person examinations.^8^

#### Residual limb care

After amputation, it is important to ensure residual limb healing and maturity before prosthetic fitting and training. This requires specialized physical therapy to assist with edema management and beginning a training program to treat or prevent contractures, improve strength and conditioning, and enhance mobility, while mitigating fall risks. Whether patients transition to inpatient care, subacute care, or home for rehabilitation during the preprosthesis training phase, specialty therapists have limited time with each patient, leaving gaps in care between sessions. Although not specific to amputation rehabilitation, Zbogar et al used wearable sensors to examine the physical activity levels of inpatients undergoing spinal cord injury rehabilitation. Researchers found that patients were rarely exercising outside of scheduled therapy sessions, despite evidence showing that more therapy could improve recovery outcomes.^32^ Using this study as a model, it may be beneficial for future research to examine how telehealth and wearable technology may be used to encourage increased physical activity and promote self‐management both within the hospital and after discharge. In addition, the wearable technology in combination with telehealth may also offer enhanced residual limb management, hastening recovery and improving the future success of prosthetic fitting and training.^33^

#### Pain management

There are a variety of pain reduction methods specifically in reference to phantom limb pain (PLP) that can be performed at home with the assistance of telehealth. Most literature focuses on mirror therapy and tactile discrimination therapy (TDT), because both of these techniques are fairly low cost and patients or family members can perform them relatively easily at home. Mirror therapy uses visual feedback to alleviate PLP by reflecting an image of the intact limb to where the phantom limb feels to be.^34^ TDT, conversely, teaches discrimination between different tactile feedback to align motor output and sensory feedback.^35^

Wakolbinger et al performed a feasibility study on TDT and found that the at‐home program had no harmful side effects or patient dropout. Additionally, despite the small sample size, preliminary evidence seemed to indicate a reduction in PLP intensity and frequency as a result of TDT treatment.^35^ Gover‐Chamlou and Tsao analyzed two case studies of at‐home mirror therapy being used to treat PLP after major amputation. In both cases, instructions were given over email by the treating physician, and patients performed mirror therapy in 4‐week segments. After completion of at‐home mirror therapy (one patient needed two 4‐week segments) PLP completely resolved in both patients.^34^ Therefore, there appear to be a variety of techniques that may be successfully performed at home or in conjunction with telehealth to improve pain management and allow patients greater autonomy in their own care.

### Prosthetic training phase

Successful prosthetic fitting and training often require an extensive amount of time and considerable practice, which may not be practical for patients who live in remote areas or who have challenges with transportation. In these cases when barriers to in‐person prosthetic training cannot be overcome, physicians, physical therapists, and other health care providers are able to identify and diagnose problems associated with the prosthesis virtually; however, any device modifications will need to be addressed by a certified prosthetist in person. We recognize this limitation while acknowledging that telehealth can improve communication and shared clinical decision making of these interdisciplinary team members, especially in rural communities. This situation is further complicated when local therapists may not possess the skills or experience in caring for individuals with amputation.^33^ Therefore, if patients were able to access high‐quality rehabilitative care either closer to their home or within their homes, it may have a significant improvement on overall outcomes. Telehealth has the potential to greatly enhance this important phase of care.

#### Residual limb and socket interface

One of the most challenging aspects of prosthetic training is achieving a comfortable and stable socket interface between the patient's residual limb and prosthesis. This is particularly difficult during the first year of recovery because of the significant fluctuations that occur in residual limb volume. Telehealth, specifically RPM, may offer considerable improvement in this aspect of care for individuals with acquired amputation, as wearable systems may provide real‐time objective data to patients and providers regarding residual limb health. Sanders et al utilized a wearable bioimpedence analyzer to objectively assess residual limb volume and its correlate with patient activity. They found that the time spent weight bearing may not be the only contributor to limb volume changes throughout the day.^36^ Additional studies have also examined the use of wearable sensors to assess patient‐socket interface, including the displacement of a limb within a socket, also known as pistoning. Sensors were embedded within the socket,^37^ or through ferromagnetic targets in a socket liner,^38^ and provided proof of concept that such systems may play an important role in future prosthetic fitting and adjustments.

#### Gait training

Proficiency in lower limb prosthetic use requires extensive gait and balance training. Telehealth may offer a novel approach to not only improve the efficacy of this aspect of rehabilitation but also to provide a more engaging experience for the patient, thus enhancing their motivation and compliance. A systematic review of rehabilitation, not specific to individuals with amputations, found that the use of certain mHealth apps had a positive effect on functional outcomes such as gait, mobility, and self‐management skills.^39^ One study examined the application of machine learning to mHealth to provide real‐time detection of falls in participants with amputation, to trigger immediate care assistance.^40^ Their findings reported only two false alarms per day from their machine learning mHealth model, as opposed to 122 false alarms with the control model.^40^ If these types of devices can be implemented into the routine care of individuals with limb loss at high risk for falls, it may allow patients and family members greater freedom, security, and quality of life.

Other studies have used various types of monitoring technology to ensure patient adherence to therapy. One study tested whether a specifically targeted behavior‐change intervention via a weekly telephone call could result in better physical functioning and walking capacity for individuals with vascular‐related amputation.^41^ Although this particular behavior‐change intervention did not improve any of the physical function measures, it did increase walking activity among participants.^41^ Other researchers have taken different approaches to target interventions through remote monitoring. Two such studies used tracking features on the participant's shoes. The first was a smartphone app that was used in conjunction with inertial measurement sensors attached to the outside of the shoes of individuals with Parkinson disease.^42^ Individuals improved more in gait speed and balance in the intervention group than in the control group, which used conventional home‐based gait training.^42^ The second study utilized a shoe insert and ankle mounted box to provide real‐time gait feedback in individuals with no gait abnormalities. This system was able to produce gait asymmetry in healthy adults despite a relatively short training period and thus may be able to produce the opposite effect in individuals with lower limb amputations.^43^

### Lifelong care phase

Once patients with amputation have achieved successful prosthetic fitting and training, the main goals for lifelong care include continued mobility and independence, sustainable and successful pain management techniques, and continuing support of their psychosocial needs. After the first year of the rehabilitation, however, it should be recognized that individuals with limb loss often receive less direct health care and rehabilitation, which may be further complicated if the patient relocates to a more remote geographical area. Telehealth offers unique opportunities for providing ongoing health care interventions at regular intervals, unencumbered by geographical barriers.

#### Mobility continuation

Imam et al used a combination of telehealth and self‐directed home exercise programs to improve mobility in older adults with lower limb amputation. Researchers found improvements in walking capacity in the intervention group that used an exergame or a video game that requires exercise. The control group, conversely, utilized a video game while sitting, and their walking capacity declined.^44^ The exergame utilized in this study was an active video that helped participants practice yoga, balance activities, strength training, and aerobic exercises, whereas the control video game was played while sitting and did not require much physical movement beyond the use of their hands. Although efficacy is important, it does not have much consequence unless there is high patient adherence. Imam et al found that in‐home adherence to the rehabilitation program, in both the intervention and control groups, was lower than when the training took place in the clinic.^44^ In a feasibility study paired with this same framework, Tao et al compared supervised and unsupervised training in the home via telehealth. Similar to the results of Imam et al, researchers found that participants used the exergame less frequently when they were unsupervised in their home.^45^ That being said, despite a decrease in frequency, the duration of this training was therapeutically sufficient.^45^ Therefore, although there is promising evidence that telehealth can be used effectively in the home, participant adherence appears to be relatively low. Future studies should explore how to utilize telehealth to encourage active lifestyles and continued adherence.

#### Sustainable pain management

Rothgangel et al performed a randomized controlled trial to examine the effects of mirror therapy on PLP, with and without telehealth. A total of 75 participants with unilateral lower limb amputation were randomized to receive 4 weeks of traditional mirror therapy followed by a virtual care mirror therapy, 4 weeks of traditional mirror therapy followed by self‐delivered mirror therapy at home, or 4 weeks of sensorimotor exercises without a mirror followed by self‐delivered exercises. All participants had an approximate average of 3 years since amputation.^46^ The researchers found that mirror therapy treatment led to an average reduction in pain intensity by 26.3%, as opposed to only 6.9% in the control treatment of sensorimotor exercises. Mirror therapy via telehealth was more beneficial than the control treatment but did not have a significant effect on pain reduction compared to the traditional mirror therapy group. The authors attributed this to a lack of embodiment of the amputated limb in the virtual environment, though future research is needed to confirm this hypothesis.^46^ Additionally, in a simultaneous feasibility study, Rothgangel et al found that more patients (56%) met clinical framework guidelines for the telehealth protocol than the traditional mirror therapy protocol (31%).^47^ Thus, it seems as though there is more patient engagement with the telehealth model than with traditional mirror therapy, but further research needs to be done to ensure that the quality of care and outcomes remain high. Therefore, although patient adherence to telehealth treatment is perhaps higher than traditional in‐person care, there is conflicting evidence as to whether telehealth treatments are as effective. Thus, future research should continue to study the efficacy of telehealth and how to ensure that this desired modality is constructive for patients' rehabilitation process.

#### Psychosocial needs

Recent studies demonstrate that having a close network of peers during the rehabilitative process who share similar experiences can improve motivation, compliance, and long‐term health outcomes.^48^ Although support groups can be very helpful in augmenting rehabilitation efforts, they face several challenges, especially for patients with geographical barriers.^49^ Telehealth has the potential for overcoming those barriers by allowing greater connection of individuals with similar injuries, interests, or life experiences through virtual group meetings or even recorded messaging. This may be particularly true for individuals with limb loss, as one study found that 100% of surveyed individuals with amputation reported that technology would improve participation in support groups.^49^ In addition to peer support, evidence suggests that telephone‐based counseling may also help individuals with physical disabilities, who may be experiencing difficulty with coping, community integration, and depression.^6^ Another case study examined the Un*limb*ited Wellness program, which was an online, or virtual care, support group for individuals with upper limb loss or difference. The program's mission was to “mitigate challenges, offer resources to improve understanding of secondary conditions, learn strategies for self‐advocacy, and access peer support” for these individuals.^50^ After the 12‐week course, individuals from a focus group reported increased scores in physical, mental and emotional, and spiritual health as well as embracing new behaviors that helped them feel empowered through their care.^50^

## CONCLUSIONS

The advancement in platforms available for telehealth and telerehabilitation have great potential for improving the care for individuals with amputation. Whether instituting limb preservation strategies, addressing wound management, promoting prosthetic fitting and training, or supporting the psychosocial challenges with acquired limb loss, evidence supports the complementary role of telehealth across the entire spectrum of care for this particularly vulnerable population, that also experiences significant health care disparities.^51^ Although telehealth has many potential benefits, it is also important to consider the need for continued “in‐person” care, which includes building key relationships between providers and patients, hands‐on modifications to technique or devices by providers and clinicians, the ability for patients to access the entirety of the care team on a regular basis, and the importance of a network of peers going through the same care experience.^48^ Although these principles can be applied to various telehealth settings, telehealth interventions should ideally be incorporated as a complementary tool within a comprehensive holistic approach to caring for individuals with limb loss. The COVID‐19 pandemic has served as a catalyst for advances in virtual care, with applications in all areas of medicine, including rehabilitation. When used appropriately, we believe that telehealth has the capacity to greatly improve access to care, patient participation, and overall outcomes. The major limitation of this study is the dearth of literature on individuals with amputations receiving virtual care. The authors were required to extrapolate results from non‐limb loss populations because these studies do not yet exist. We recognize this paucity of research and therefore felt that these studies were beneficial to the reader and added more information. Although further research is needed in the limb loss population, the current literature is certainly promising and serves as a strong foundation for future innovations and technologically advanced systems used in the care of individuals with amputation.

## CONFLICT OF INTEREST

The authors have no conflicts of interest to disclose.

## DISCLAIMER

The views expressed in this publication are those of the authors and do not reflect the official policy of the Department of Defense, the U.S. Government, the Uniformed Services University, or the Henry M. Jackson Foundation.
